# Thinking about the environment and theorising change: how could Life History Strategy Theory inform mHealth interventions in low- and middle-income countries?

**DOI:** 10.1080/16549716.2017.1320118

**Published:** 2017-06-15

**Authors:** Barak Morgan, Xanthe Hunt, Mark Tomlinson

**Affiliations:** ^a^ Global Risk Governance Programme, Institute for Safety Governance and Criminology, Law Faculty, University of Cape Town, Rondebosch, South Africa; ^b^ NRF Centre of Excellence in Human Development, DVC Research Office, University of Witwatersrand, Johannesburg, South Africa; ^c^ Department of Women’s and Children’s Health, Neonatal Unit, Karolinska Institute, Stockholm, Sweden; ^d^ Department of Psychology, Stellenbosch University, Stellenbosch, South Africa

**Keywords:** Life History Strategy Theory, mHealth, interventions, public health

## Abstract

**Background**: There is a growing body of literature outlining the promise of mobile information and communication technologies to improve healthcare in resource-constrained contexts.

**Methods**: We reviewed the literature related to mobile information and communication technologies which aim to improve healthcare in resource-constrained contexts, in order to glean general observations regarding the state of mHealth in high-income countries (HIC) and low- and middle-income countries (LMIC).

**Results**: mHealth interventions in LMIC often differ substantively from those in HIC, with the former being simpler, delivered through a single digital component (an SMS as opposed to a mobile phone application, or ‘app’), and, as a result, targeting only one of the many factors which impact on the activation (or deactivation) of the target behaviour. Almost as a rule, LMIC mHealth interventions lack an explicit theory of change.

**Conclusion**: We highlight the necessity, when designing mHealth interventions, of having a theory of change that encompasses multiple salient perspectives pertaining to human behaviour. To address this need, we explore whether the concept of Life History Strategy could provide the mHealth field with a useful theory of change. Life History Strategy Theory may be particularly useful in understanding some of the problems, paradoxes, and limitations of mHealth interventions found in LMIC. Specifically, this theory illuminates questions regarding ‘light-weight’ programmes which solely provide information, reminders, and other virtual ‘nudges’ that may have limited impact on behaviours governed by extrinsic structural factors.

## Background

There is a growing body of literature outlining the promise of mobile information and communication technologies to improve healthcare in resource-constrained contexts [1]. These interventions, usually referred to as mobile health or mHealth, have received significant support from policymakers and donors interested in their potential to reach underserved populations [2–5].

Substantive claims have been made about the potential of mHealth interventions in low- and middle-income countries (LMIC) [1,6–14] where broad access to mobile phone technologies appears to present a unique opportunity to improve healthcare service delivery in contexts where health services and infrastructure are limited [1]. However, even a brief examination of the mHealth literature from LMIC and high-income countries (HIC), respectively, reveals discrepancies between the types of interventions in these two contexts, as well as in terms of the evidence produced. One of the most striking differences is that those in LMIC lack a coherent theory of change (TOC).

The importance of grounding health interventions in behaviour change theory has been previously described [15–18]. It has been argued that human behaviour remains the largest source of variance in health-related outcomes [15,19]. People’s health is affected by numerous lifestyle factors, many of which are determined by controllable and often avoidable behaviours [19]. Yet, people find it hard to change, particularly, it seems, if the change involves behaviours associated with the greatest health risks (for instance, smoking, drinking, or eating fatty foods). Interventions which strive to help people move towards healthful changes, then, often face challenges [15].

Understanding what processes might underlie human behaviour in this domain may make it possible for interventions to better target aspects such as the environment, and intra- or extra-personal characteristics, that are most likely to influence these processes. A coherent TOC might be expected to guide interventionists’ expectations for health-promoting programmes, better target interventions to those most likely to benefit, and refine intervention strategies to address relevant mediators and moderators of the target behaviour [20]. Given current debates about investing in mHealth, working within a conceptual framework may make it easier to design interventions that are more likely to work because they attend to facets of the individual’s world most likely to influence a given behaviour.

In light of this, we propose Life History Strategy Theory (LHST) as a heuristic TOC which helps to conceptualise the mechanisms whereby brains, environments, and mHealth interventions interact to bring about behaviour change. We propose that this theory is particularly useful for thinking about behaviour change in LMIC, as it attends to facets of the environment proposed, in past work, to be particularly salient to the regulation of behaviours in these settings. This way of thinking about behaviour change, we suggest, may account for why some types of mHealth interventions appear to be more successful in HIC than in LMIC.

## Scoping of the literature

In order to examine the discrepancies between the mHealth research from LMIC and HIC, we conducted a brief review of the peer-reviewed literature, and consolidated past systematic reviews in the field. The aim of our search was not intended to be a systematic review but rather to gain a rapid overview of where the field is currently. In our review, we assessed papers on a number of dimensions (see Table 1).

**Table 1.** Literature review.

Component	Category	HIC papers (%)	LMIC papers (%)
Component types in intervention package	Digital only	79	63
	Digital + resource	21	36
Setting (proxy for environmental conditions and resources)	Lower income	15	81
	Higher income	85	19
Complexity of digital component	Interactive app	61	19
	Simple messaging	39	81
Theory of Change	Theoretical	48	19
	Implicit but not explicated	33	25
	None	19	56

Note: For a comprehensive list of the articles included in this review, please see the Appendix.

We searched Google Scholar, Academic Search Premier, PsycARTICLES, and ProQuest Social Science Journals for the search terms ‘mHealth’, ‘mobile health’, ‘e-health’, and ‘telemedicine’. The search yielded 149 papers. The papers were then examined for relevance. Those which were theoretical, protocols, or other article formats aside from interventions, were excluded. Sixteen articles concerning interventions from LMIC were included, and 33 from HIC. We also reviewed four past systematic reviews of mHealth intervention literatures [2,21–23].

We begin the following section with a discussion of these systematic reviews. Thereafter, we discuss the evidence gleaned from our search which, although not a systematic account of the literature, reveals a broad outline of the differences in mHealth between low- and middle-income (LAMI) and HIC contexts.

## Literature review

Several systematic reviews have examined the mHealth intervention literature, both globally [2], and in LMIC in particular [21–23]. Free et al. [2] identified 75 trials, of which 59 were interventions to improve disease management and 26 were interventions to change health behaviours (disease prevention). All the trials that focussed on health behaviour change were conducted in HIC. In their review, only seven interventions included a TOC, although a number included theoretically driven behaviour-change techniques. The authors’ key recommendations included the need for high-quality, adequately powered trials of interventions which would allow for better evaluation of effects on objective outcomes.

Beratarrechea et al. [23] examined controlled studies evaluating mHealth voice and text message interventions for chronic diseases in adults. They concluded that mHealth interventions for chronic diseases positively impacted on patient outcomes, improved treatment attendance, and improved Health-Related Quality of Life . However, a relatively small number of studies were included in the review, and within the studies, a relatively small number of patients enrolled. The review also focussed only on disease management, as opposed to preventative health behaviour change.

In a more recent, field-specific, systematic examination of mHealth interventions, Lee et al. [21] reviewed the mHealth literature on maternal, newborn, and child health in LMIC. Only 15 articles and 2 conference abstracts met the authors’ inclusion criteria. Of these 15, only 2 studies were graded as being at low risk of bias, and only 1 study demonstrated an improvement in morbidity or mortality. The authors concluded that the majority of mHealth studies in the realm of maternal and child health in LMIC were of very poor methodological quality. Despite the fact that some studies reported improvements in intermediate outcomes, few evaluated impacts on user outcomes, and most did not fully explain the basis of their intervention (theoretical or otherwise) [21]. They described the mHealth projects as typically ‘under-theorised, poorly specified and vaguely described’ [21,p.14]. Thus, not only did these authors echo the call of Free et al. [2] for more rigorous studies and better evidence in the field of mHealth, but they also highlighted the lack of theoretical grounding in mHealth in LMIC.

However, in a contemporaneous review of the literature concerning the effect of mHealth interventions in improving maternal and neonatal care in LMIC, Sondaal et al. [22] examined 27 studies, of which 12 were intervention papers and 15 descriptive ones. These authors concluded that the emerging focus on strong experimental research designs, combined with government involvement and integration of mHealth interventions into the healthcare system, was a positive development, and augured well for the field, a marked departure from the conclusion of Lee et al. [21].

Concerning our own review of the literature, only one out of five interventions in HIC were comprised of digital and non-digital components, while two out of five from LMIC contained digital and non-digital components. In LMIC, the non-digital component often comprised incentive shopping vouchers or cash transfers.

The majority of high-income interventions are app-based (61%), while the vast majority of LMIC interventions are not (19%). Apps have the potential to influence how the user engages with various facets of their environments in nuanced ways [24]. Apps can provide contingent feedback related to risk behaviours in real time. For example, GPS technology can alert a recovering alcoholic to the proximity of a bar, thereby preparing the mind before the drinking cue comes into sight or helping them to avoid a potential trigger [25]. In a recent example, AndWellness [26], a mobile personal sensing application built for Android, includes a suite of mobile services and server-end software to improve personal health and wellness. AndWellness will initially focus on obesity prevention and weight management. As users go about their normal routine, the app profiles their behavioural patterns using continuous sampling of available on-board sensors (such as WiFi, Bluetooth, GPS, and accelerometer). It analyses this information and identifies triggers (for instance, if they have been sitting still for too long, or are near a restaurant). It then prompts the user to record audio, video, or images for future reflection [26]. In other cases, apps engage with sophisticated, well-resourced existing healthcare infrastructure [24].

Perhaps the most striking discrepancy between the literatures from LMIC and HIC, however, concerns TOC. Only 19% of papers reporting on mHealth interventions in LMIC contained an explicit TOC, in contrast to nearly half (48%) of those from HIC.

It is worth examining, briefly, some examples of mHealth interventions in HIC which incorporate TOCs that attend to factors which influence the behaviour the interventions are designed to target. An mHealth intervention for the management of type 1 diabetes in adolescents was explicitly designed to target decision inertia and include gamification [27]. The combination of simple automated reminders and a rewards system worked together to produce a behavioural mechanism that produced a significant positive change. The pilot evaluation of this intervention showed that blood glucose measurements increased by 50%. By incorporating elements grounded in an understanding of the target group and the target behaviour, the interventionists were able to deliver an effective intervention.

In another example, Rabbi et al. [28] report on MyBehavior, a mobile phone app designed to process tracked physical activity and eating behaviour data and provide personalised, actionable, low-effort feedback and suggestions to users, contextualised to their environment and previous behaviour. Rabbi et al. [28] note that MyBehavior is grounded in contemporary behavioural science theories including learning theory, social cognitive theory, and the Fogg Behavior Model (FBM). Knowing that there are numerous internal and external factors which determine eating and exercise behaviours, MyBehavior designers looked to numerous, complementary TOCs to guide their intervention efforts – learning theory to assess whether a person has the skills needed to perform a behaviour; the Fogg Behavior Model to guide the design of tools to prompt low-effort actions; and social cognitive theory, to develop users’ self-efficacy in order to facilitate their engagement in health-promoting behaviour. Following the pilot, MyBehavior users also walked significantly more than the control group over the three weeks of the study. Further, qualitative daily diary, interview, and survey data showed that MyBehavior users not only found the app’s behaviour suggestions to be highly actionable, but they also indicated a willingness to follow the suggestions.

In other examples, Pramana et al. [29] developed an app to support Cognitive Behavioural Therapy (CBT) for children with anxiety. The app was grounded in CBT principles, and incorporated numerous social support mechanisms, both of which are known to facilitate management of anxiety.

Waterlander et al. [30] developed a mHealth weight management programme using proven face-to-face behaviour change techniques which incorporated input from the target population. The intervention resulted in changes in body weight and body mass index (BMI) at 12 weeks, which indicated that the programme could be effective in supporting people with weight loss (although the authors did note the high dropout rate to be a concern).

However, the lack of TOCs is a conspicuous gap in mHealth intervention development in LMIC. As noted, TOCs in HIC attend to numerous facets of the intra- and extra-personal environment which interventionists have reason to believe might influence the target behaviour. It is not our intention to propose that these TOCs will or will not work in LMIC, or should be transposed from HIC to LMIC. Rather, we wish to highlight that TOCs should be considered by interventionists in LMIC. The choice of TOC to guide an intervention will differ depending on the target behaviour. Given environmental factors, which will be reflected upon in the section which follows, we propose LHST as a potential TOC which may account for some of the failures of mHealth interventions in resource-strapped contexts, and point to necessary developments to be made in the field to facilitate mHealth effectiveness.

## Life History Strategy Theory (LHST)

Based on the systematic reviews previously discussed, and our review of the literature here, no interventions of which we know drew on LHST in intervention design, or, indeed, made mention of LHST at all. This is thus the first attempt to employ LHST to critique, and inform thinking about, mHealth.

Underlying LHST is the premise that behaviour is shaped and regulated by the environment. Specifically, LHST proposes that humans, like most other species, have evolved developmental systems which respond differently and adaptively to favourable and adverse environments [31–35]. Evidence suggests that the environment an individual encounters early in life shapes their development in strategic ways that are adaptive under those conditions [32,36,37]. Three core dimensions of the environment influence development: environmental safety, harshness, and predictability [32,35–37]. In human populations, the best barometers for environmental safety are premature child and adult mortality and morbidity, while socioeconomic status is a good indicator of harshness [35,38,39]. Finally, predictability refers to the extent to which levels of safety and harshness fluctuate or remain stable.

Life history strategies (LHS) are conceived of as falling along a continuum, from ‘fast’ to ‘slow’. It is important to note that these developmental trajectories are strategically adaptive. Adverse environments steer development towards fast LHS [33,36,40]. A fast LHS encompasses rapid growth to maturity, and early reproduction yielding many offspring with relatively less parental investment per offspring. In the context of adversity, an individual should make the most of the few opportunities afforded them to reproduce in their potentially short life, and limit their investment in long-term life plans [7,33,36]. Safe, bountiful, and stable environments steer development towards a slow LHS [36]. A slow LHS entails slower growth to maturity, delayed reproduction, slower rate of reproduction, and greater parental investment in children. In contexts of abundance, it makes more sense for individuals to amass the resources and skills necessary to be competitive in a more complex and nuanced social environment.

The behaviours that characterise fast and slow LHS become deeply woven into brain structure and function, especially during early childhood [34,41]. LHST therefore suggests that humans become locked into LHS behaviours from an early age. Although there is evidence to support this [35–37], we also know that adults are capable of rapid behavioural change (for instance, interventions targeting eating behaviour [42] or medication adherence [43]), including LHS behaviours [44–46]. This behavioural plasticity (the potential to change behaviour without major reorganisation of the brain) is cued by the environmental conditions prevailing in adult life. Behavioural plasticity thus provides a window of opportunity for interventions to influence behaviour in adulthood; but crucially, unlike behaviour woven into the brain during early development when neuroplasticity is high, change that relies on behavioural plasticity will only be sustained for as long as the corresponding changes in environment prevail.

From this perspective, the power of interventions to shift behaviour towards a slow LHS, with a lower health and psychosocial risk profile, is limited by the degree and duration of change in dimensions of the environment that regulate the expression of LHS behaviours, viz. safety, harshness, and predictability.

## What LHST means for mHealth

LMIC are frequently characterised by harsh, unsafe, and unpredictable conditions which regulate for fast LHS. Fast LHS, while adaptive in adverse environments, also entail behaviours associated with increased health and psychosocial risks for parents and children. These include early pregnancy, aggressive and frequently violent male–male competition, impulsive decision-making, and lower parental investment (e.g. in healthcare and education) [47]. Another core feature of fast LHS is future-discounting, meaning short-term gains are given priority over long-term goals [47]. Future-discounting may be a particular challenge to interventions aimed at reducing behaviours associated with long-term health risks such as smoking and at adherence to tuberculosis (TB) or HIV treatment. In sum, mHealth interventions which target LHS-relevant behaviours in LMIC settings face the challenge of working directly against environmental conditions regulating behaviour in the opposite direction [47].

Particular shortcomings of mHealth interventions in LMIC include their failure to attend to the fact that harsh, unpredictable, and resource-strapped environments may hinder positive behaviour change. LHST provides a way of conceptualising this failure by drawing our attention to environmental constraints on certain health behaviours, and directing us to think about how behaviour change can be sustained in the face of environmental challenges. In short, in LMIC, environments may come to exert more of an influence on human behaviour (there are very real and immediate ways in which environmental factors like harshness impact on the daily lives of persons in these settings) than in HIC. As such, it is imperative that a TOC for mHealth interventions in LMIC attend to the environment.

LHST presents a fundamental challenge to the widespread use of mHealth interventions in LMIC, as digital interventions alone are unlikely to effect real, sustained context change. We could conceive of digital interventions as virtual ‘nudges’ towards the target behaviour – virtual because they do not actually change the environment. According to LHST, virtual nudges are not enough to change health behaviour (e.g. behaviours that reduce long-term health risks) when that behaviour is being strongly regulated in the opposite direction (e.g. future-discounting) by life-history-relevant dimensions of the environment. Notably, not all health behaviours are determined by LHS-relevant facets of the environment. Recalling our earlier discussion of past reviews, it is noteworthy that Beratarrechea et al. [23] showed that mHealth might be effective for chronic disease management, as opposed to Lee et al.’s [21] finding regarding health behaviour change amongst mothers. Indeed, from an LHST perspective, these findings may make sense. Whilst maternal behaviour (which mHealth interventions seem to minimally impact) is an LHST-relevant behaviour, chronic disease management may not be as susceptible to the regulating effects of LHS-relevant environmental forces.

In the case of LHS-relevant behaviour, the following must be noted. Recalling our observation earlier that behaviour change in adulthood depends on behavioural plasticity (which is cued by the environmental conditions prevailing in adult life), positive behaviour change in adulthood requires that the facilitative environmental changes be sustained in order for the target behaviour to be sustained. LHST would suggest that, in order to change behaviour, the digital component of mHealth interventions, the virtual nudge, must work hand in hand with an environment that is inherently sufficient or has as a consequence of non-digital components of the intervention been rendered sufficient to facilitate the behavioural change and sustain it.

Effecting the change entails basing a given intervention on a TOC which accounts for the relevant environmental conditions which influence the target behaviour. Effecting and sustaining the change involves exploiting behavioural plasticity by sustaining the environmental changes that facilitate the target behaviour.

LHST can potentially inform the design of mHealth interventions. Theoretical principles to consider include:

- Is the target behaviour susceptible to environmental regulation? If so, what dimension(s) of the environment pertain?
- For relevant environmental dimension(s), what features/resources can be sustainably leveraged or changed? (E.g. are there social/health services readily available to support behavioural nudges encouraged by the mHealth intervention? Can community unsafety engendering fast LHS behaviours be addressed?)
- How will the mHealth intervention work in relation to the environment? What sort of digital mHealth intervention is needed (simple messaging or complex app)?
- What non-digital intervention components might be necessary?
- Is the non-digital intervention sustainable over time?
- LHS behaviours are associated with both short-term and long-term risks. Evidence of long-term efficacy requires long-term follow-up.

In our survey of the literature, we found that mHealth interventions in LMIC were more likely to lack an explicit TOC, more likely to comprise a simple messaging digital component, and more likely to include a non-digital component. In contrast, mHealth interventions in HIC were more likely to be purely digital and to be app- rather than SMS-based, and more likely to engage with existing well-resourced and often sophisticated healthcare infrastructure in the users’ environment [48,49].

In sum, mHealth in HIC constitutes virtual digital ‘nudges’ which are more likely to succeed because they connect the user with a well-resourced environment that is sufficiently safe, favourable, and predictable to regulate for slow LHS behaviours associated with long-term thinking. In LMIC, where the environment is often regulating behaviour in the direction opposite to that desired by the interventionists, such nudges are unlikely to work. The fact that LMIC mHealth interventions often include non-digital components such as shopping vouchers or cash transfers suggests a belief amongst interventionists of the need to incentivise behaviour change beyond that which is achievable through mere messaging, as may work in HIC settings. However, when this incentivisation is not grounded in a TOC, it is difficult to design, predict, and assess its impact. For example, are shopping vouchers and cash transfers conceived as incentives or as interventions that meaningfully address a harsh socioeconomic environment? According to LHST, only the latter is likely to impact behaviour and then only for as long as the cash transfer intervention is sustained.

Given that LHST is not only an evolutionary theory, but also one which places emphasis on the environmental factors which influence behaviour, there is no reason to believe that it would be difficult to include LHST in intervention design. It is also not mutually exclusive with other theories of behaviour change. For instance, if a behaviour change intervention were to use the principles of social cognitive theory to motivate the incorporation of social support in an mHealth intervention to decrease depression and improve infant care amongst mothers in low-income communities, it would simultaneously be attending to and altering environmental harshness and unpredictability, both of which are, according to LHST, important determinants of wellbeing and parental investment. The benefit of LHST lies primarily in the fact that it draws our attention to the facets of adverse environments which, if left unattended, are unlikely to be conducive to positive behaviour change.

Important future directions for the field of mHealth will be the examination of where, and when – in which contexts and with which behaviours – the principles of LHST are of most import for designing mHealth interventions. Naturally, mHealth interventions have been used for a range of different conditions in LMICs, and for different behaviours. As noted, some behaviours are more susceptible to LHS-relevant facets of the environment than are others (recall our discussion of chronic disease management versus maternal investment).

Future, rigorously evaluated, programmes could usefully explore whether such LHS-relevant behaviours are better activated, and longer maintained, under altered environmental conditions, versus simply as a result of an mHealth intervention alone. Comparing, for instance, ‘cash plus care’ (the care being an mHealth intervention) [50] versus a simple mHealth intervention for parenting, could provide tentative evidence for some of the tenets of LHST if the behaviour change were (a) to be greater for the cash plus care condition, and (b) to be sustained only as long as the cash intervention continued.

## Limitations

A limitation of the present commentary is the nature of our literature review, which was conducted with the aim of gaining a rapid overview of where the field is currently, rather than providing a systematic account of mHealth work.

## Conclusions

mHealth has enormous potential to improve health and development in LMIC, but to realise this potential it needs to be part of broader integrated packages of care across different platforms – packages which address those dimensions of the environment that regulate behavioural plasticity. Currently, however, mHealth in LMIC risks being marginalised as a result of its over-reliance on interventions comprising only virtual nudges coupled with a near-universal lack of explicit TOCs.

There is a considerable body of research detailing effective face-to-face interventions for facilitating positive health behaviour, and much of this literature suggests multifaceted interventions are required to change behaviour [2]. Developing and evaluating theory-driven interventions which attend to relevant influencers of behaviour is key to ensuring mHealth delivers on some of its promises. We believe that LHST offers important ways to conceptualise mHealth interventions that could be usefully employed to ensure improved effectiveness outside of small, short-term pilot studies.

## Paper context

This article explores the use of Theory of Change (TOC) in the mHealth literature pertaining to health behavior interventions across high (HIC) and low/middle (LMIC) income settings. We observed that only one-fifth of mHealth studies in LMIC included an explicit TOC whereas in HIC half did. We also noted that environmental regulators of health behavior differ markedly across income settings with LMIC settings more likely to be characterized by environmental adversity in three domains: harshness, lack of safety, and unpredictability. According to Life History Strategy Theory (LHST), adverse environmental conditions in these domains are likely to regulate (push) behavior in the opposite direction to that the mHealth intervention is aiming to achieve. Failure to incorporate LHST into mHealth interventions in LMIC settings may therefore leave mHealth interventions lacking. This article demonstrates the potential utility of explicitly including a TOC in mHealth research.

## AppendixThe list of papers included in this review:

Aikens JE, Zivin K, Trivedi R, Piette JD. Diabetes self-management support using mHealth and enhanced informal caregiving. J Diabetes Complications 2014;28(2):171–176.

Baird SJ, Garfein RS, McIntosh CT, Özler B. Effect of a cash transfer programme for schooling on prevalence of HIV and herpes simplex type 2 in Malawi: a cluster randomised trial. Lancet 2012;379(9823):1320–1329.

Chaiyachati KH, Loveday M, Lorenz S, Lesh N, Larkan LM, Cinti S, et al. A pilot study of an mHealth application for healthcare workers: poor uptake despite high reported acceptability at a rural South African community-based MDR-TB treatment program. PLoS One 2013;8(5):e64662.

Chang LW, Kagaayi J, Arem H, Nakigozi G, Ssempijja V, Serwadda D, et al. Impact of a mHealth intervention for peer health workers on AIDS care in rural Uganda: a mixed methods evaluation of a cluster-randomized trial. AIDS Behav 2011;15(8):1776–1784.

da Costa TM, Barbosa BJ, e Costa DA, Sigulem D, de Fátima Marin H, Castelo Filho A, et al. Results of a randomized controlled trial to assess the effects of a mobile SMS-based intervention on treatment adherence in HIV/AIDS-infected Brazilian women and impressions and satisfaction with respect to incoming messages. Int J Med Inform 2012;81(4):257–269.

De Walque D, Dow WH, Nathan R, Abdul R, Abilahi F, Gong E, et al. Incentivising safe sex: a randomised trial of conditional cash transfers for HIV and sexually transmitted infection prevention in rural Tanzania. BMJ Open 2012;2(1):e000747.

Fedha T. Impact of mobile telephone on maternal health service care: a case of Njoro Division. Open J Prev Med. 2014;4(5):365–376.

Fotso JC, Robinson AL, Noordam AC, Crawford J. Fostering the use of quasi-experimental designs for evaluating public health interventions: insights from an mHealth project in Malawi. Etude Popul Afr 2015;29(1):1607.

Ginsburg OM, Chowdhury M, Wu W, Chowdhury MT, Pal BC, Hasan R, et al. An mHealth model to increase clinic attendance for breast symptoms in rural Bangladesh: can bridging the digital divide help close the cancer divide? Oncologist 2014;19(2):177–185.

Gustafson DH, McTavish FM, Chih MY, Atwood AK, Johnson RA, Boyle MG, et al. A smartphone application to support recovery from alcoholism: a randomized clinical trial. JAMA Psychiatry 2014;71(5):566–572.

Heffner JL, Vilardaga R, Mercer LD, Kientz JA, Bricker JB. Feature-level analysis of a novel smartphone application for smoking cessation. Am J Drug Alcohol Abuse 2015;41(1):68–73.

Lund S, Hemed M, Nielsen BB, Said A, Said K, Makungu MH, et al. Mobile phones as a health communication tool to improve skilled attendance at delivery in Zanzibar: a cluster-randomised controlled trial. BJOG 2012;119(10):1256–1264.

Lund S, Nielsen BB, Hemed M, Boas IM, Said A, Said K, et al. Mobile phones improve antenatal care attendance in Zanzibar: a cluster randomized controlled trial. BMC Pregnancy Childbirth 2014;14(1):1.

Mbuagbaw L, Thabane L, Ongolo-Zogo P, Lester RT, Mills EJ, Smieja M, et al. The Cameroon Mobile Phone SMS (CAMPS) trial: a randomized trial of text messaging versus usual care for adherence to antiretroviral therapy. PloS One 2012;7(12):e46909.

Montes JM, Medina E, Gomez-Beneyto M, Maurino J. A short message service (SMS)-based strategy for enhancing adherence to antipsychotic medication in schizophrenia. Psychiatry Res 2012;200(2):89–95.

Park LG, Howie-Esquivel J, Chung ML, Dracup K. A text messaging intervention to promote medication adherence for patients with coronary heart disease: a randomized controlled trial. Patient Educ Couns 2014;94(2):261–268.

Pop-Eleches C, Thirumurthy H, Habyarimana JP, Zivin JG, Goldstein MP, De Walque D, et al. Mobile phone technologies improve adherence to antiretroviral treatment in a resource-limited setting: a randomized controlled trial of text message reminders. AIDS 2011;25(6):825.

Rodrigues R, Shet A, Antony J, Sidney K, Arumugam K, Krishnamurthy S, et al. Supporting adherence to antiretroviral therapy with mobile phone reminders: results from a cohort in South India. PloS One 2012;7(8):e40723.

Rubinstein A, Miranda JJ, Beratarrechea A, Diez-Canseco F, Kanter R, Gutierrez L, et al. Effectiveness of an mHealth intervention to improve the cardiometabolic profile of people with prehypertension in low-resource urban settings in Latin America: a randomised controlled trial. Lancet Diabetes Endocrinol 2016;4(1):52–63.

Thirumurthy H, Lester RT. M-health for health behaviour change in resource-limited settings: applications to HIV care and beyond. Bull World Health Organ 2012 May;90(5):390–392.

Wakadha H, Chandir S, Were EV, Rubin A, Obor D, Levine OS, et al. The feasibility of using mobile-phone based SMS reminders and conditional cash transfers to improve timely immunization in rural Kenya. Vaccine 2013;31(6):987–993.

Zurovac D, Sudoi RK, Akhwale WS, Ndiritu M, Hamer DH, Rowe AK, et al. The effect of mobile phone text-message reminders on Kenyan health workers’ adherence to malaria treatment guidelines: a cluster randomised trial. Lancet 2011;378(9793):795–803.
